# Intensive behavioural interventions based on applied behaviour analysis for young children with autism: An international collaborative individual participant data meta-analysis

**DOI:** 10.1177/1362361320985680

**Published:** 2021-01-22

**Authors:** Mark Rodgers, Mark Simmonds, David Marshall, Robert Hodgson, Lesley A Stewart, Dheeraj Rai, Kath Wright, Esther Ben-Itzchak, Svein Eikeseth, Sigmund Eldevik, Hanna Kovshoff, Iliana Magiati, Lisa A Osborne, Phil Reed, Giacomo Vivanti, Ditza Zachor, Ann Le Couteur

**Affiliations:** 1 University of York, UK; 2 University of Bristol, UK; 3 Ariel University, Israel; 4 Oslo Metropolitan University, Norway; 5 University of Southampton, UK; 6 University of Western Australia (UWA), Australia; 7 Swansea Bay University Health Board, UK; 8 Swansea University, UK; 9 Drexel University, USA; 10 Tel Aviv University, Israel; 11 Newcastle University, UK

**Keywords:** applied behaviour analysis, autism spectrum disorder, autism, individual participant data, meta-analysis, systematic review

## Abstract

This individual participant data meta-analysis is the most intensive possible evaluation of the effectiveness of early intensive applied behaviour analysis–based interventions for pre-school autistic children compared with treatment as usual/eclectic interventions. Data from 491 participants (originally collected in 10 studies) were included. Children receiving early intensive applied behaviour analysis–based interventions improved more on the Vineland adaptive behaviour scale (MD = 7.00; 95% confidence interval = 1.95–12.06) and cognitive ability (intelligence quotient) (MD = 14.13; 95% confidence interval = 9.16–19.10) relative to comparators at 2 years; though effects varied considerably across studies. Evidence for other outcomes was inconclusive due to insufficient evidence and there were few data on longer-term effects. All studies were at risk of bias across several domains, often due to the lack of randomisation or blinding of outcome assessors. Given the emerging evidence of modest, albeit short term, effects of a range of pre-school autism interventions and the limitations of the quality of evaluation studies to date, future research should investigate which supports and interventions are most effective for children and families prioritising outcomes measures that are meaningful for the autism community and longer-term follow-up. Further systematic reviews of the existing evidence are unlikely to add to the findings presented here.

**Lay abstract:**

Early intensive applied behaviour analysis–based interventions are designed to support young autistic children’s learning and development. Unfortunately, the available evidence about the effectiveness of these interventions remains unclear. Several reviews have focused on the published findings rather than contacting the authors to collect and analyse data about the individual participants in the original studies. Also, most of the studies were carried out by groups involved in delivering the interventions leading to the potential bias in interpreting the results. Our research team (supported by an international advisory group) carried out an independent individual patient data review by collecting the original participant data from the authors of the studies, to examine the effectiveness of these interventions. The results suggested that early intensive applied behaviour analysis–based interventions might lead to some changes in children’s cognitive ability (intelligence quotient) and everyday life skills after 2 years, compared with standard treatments. However, all the studies had problems with the way they were designed. Also, few of the studies looked at outcomes that have been described as most important to autistic people or followed children beyond 2 years. We think that further systematic reviews of the existing evidence are unlikely to add to the findings of our review. Furthermore, we recommend that future research should investigate which types of supports and interventions are most effective for children and families, prioritising outcomes measures that are meaningful for the autism community and include, wherever possible, longer-term follow-up.

## Introduction

Autism spectrum disorder (henceforth referred to as ‘autism’) is a spectrum condition in which individual presentation is usually a combination of social, communication and behavioural difficulties, differences and strengths, which vary considerably between individuals and over time. Autism currently has significant economic and social impacts for individuals, their families and wider society (Buescher et al., 2014; Howlin & Moss, 2012). Effective supports and interventions, targeting core developmental skills that are important for learning and independence and that support children before they reach school age, could have considerable benefits (Howlin et al., 2009; Reichow & Wolery, 2009).

Early intensive behavioural intervention (EIBI) for autism, first described by Lovaas (1987), and usually delivered on a one-to-one basis for 15–50 h per week, is based on the principles of applied behaviour analysis (ABA). These principles include a range of techniques, such as breaking down a complex skill into component parts and then teaching those parts in combination with a reward system. The techniques emphasise stimulus discrimination, learning and positive reinforcement, with the aim of shifting the child to a more positive developmental trajectory at an earlier stage (Lovaas, 1987).

Subsequent adaptations of the original model have incorporated EIBI techniques within a more naturalistic and developmentally informed framework. Known collectively as naturalistic developmental behavioural interventions (NDBIs) (Schreibman et al., 2015), they include child-led and incidental teaching. Prominent examples of models incorporating NDBI techniques include pivotal response treatment (PRT) (Koegel et al., 1999) and the early start Denver model (ESDM) (Rogers & Dawson, 2010). In the meta-analyses, we use ‘early intensive ABA-based interventions’ as an umbrella term including both EIBI and NDBI approaches.

Several systematic reviews to date have compared early intensive ABA-based interventions with treatment as usual (TAU) or other therapies (Eikeseth, 2009; Eldevik et al., 2009; Howlin et al., 2009; Makrygianni & Reed, 2010; Peters-Scheffer et al., 2012; Reichow et al., 2014; Reichow & Wolery, 2009; Spreckley & Boyd, 2009; Virués-Ortega, 2010; Waddington et al., 2016; Warren et al., 2011). Most focused either on EIBI (Eikeseth, 2009; Eldevik et al., 2009; Howlin et al., 2009; Makrygianni & Reed, 2010; Ona et al., 2020; Peters-Scheffer et al., 2012; Reichow et al., 2014; Reichow & Wolery, 2009; Spreckley & Boyd, 2009; Virués-Ortega, 2010; Warren et al., 2011), NDBI (Ona et al., 2020; Tiede & Walton, 2019) or ESDM alone (Waddington et al., 2016). Despite the approach taken by these authors, these different models of interventions share many of the same components and are often used interchangeably in ABA-based provision in the United Kingdom (Rodgers et al., 2020).

In terms of investigating moderators of intervention effectiveness, some previous reviews have considered child characteristics such as age, cognitive ability (intelligence quotient (IQ)), adaptive behaviour or verbal ability at intake, as possible moderators (Howlin et al., 2009; Makrygianni & Reed, 2010; Peters-Scheffer et al., 2012; Virués-Ortega, 2010; Warren et al., 2011). These analyses were based on limited summary/aggregate data (AD) extracted from study publications, an approach that is limited in its ability to uncover the impact of child-level characteristics, especially relevant in samples of autistic individuals presenting with a broad range of skills and needs. An alternative methodology – individual participant data meta-analysis (IPD-MA), which involves the collection and re-analysis of the original trial data sets, can more effectively study the impact of these variables (2005). One review, carried out 10 years ago, made a limited attempt to apply this methodology and examine potential effect modifiers in more detail (Eldevik et al., 2009). However, the authors considered only four data items (age, IQ and adaptive behaviour scores at intake and after 2 years) and ultimately were not able to conduct an analysis of moderator variables due to the limited number of included studies and variables at that time.

**Figure 1.**
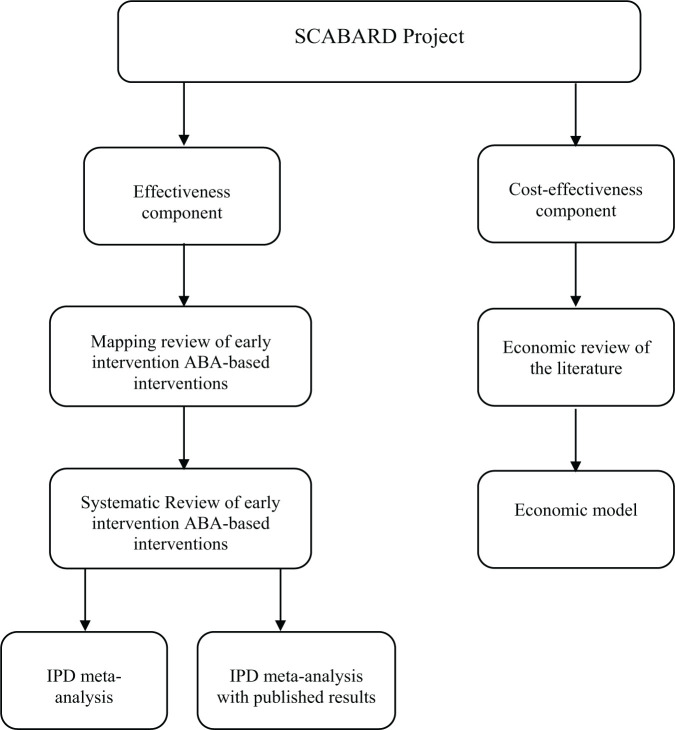
Diagram of the components of the full SCABARD project as presented in Rodgers et al. (2020).

Given the limitations of previous systematic reviews, the UK National Institute for Health Research (NIHR) funded a systematic review with an IPD-MA and economic evaluation of the effects of early intensive ABA-based interventions. SCABARD (synthesising comprehensive applied behaviour analysis interventions – research for children with autism spectrum disorders) was designed as an international collaborative partnership between study investigators who have carried out eligible primary studies and an IPD-MA research team responsible for collecting and analysing the data (Figure 1). This team was supported by an international study advisory group comprising experts by experience (caregivers of children with an autism diagnosis both with and without firsthand experience of ABA-based early interventions), autistic adults, a representative of a UK autism charity, UK-based ABA/EIBI practitioners, an IPD research specialist, together with international and UK experts from psychiatry, and clinical and educational psychology.

Figure 1 shows the various components of the SCABARD project. The final report (Rodgers et al., 2020) and future publications will provide further details including a detailed examination of individual theoretical models and an economic evaluation. This article summarises the findings from the main IPD-MAs on the effectiveness of early intensive ABA-based interventions compared with TAU or eclectic interventions.

## Methods

SCABARD followed a protocol registered on PROSPERO (CRD42017068303). Findings are reported in accordance with the PRISMA-IPD (preferred reporting items for systematic reviews and meta-analysis individual participant data) statement (Stewart et al., 2015).

### Selection criteria

Selection criteria were developed in conjunction with the advisory group. Early intensive ABA-based interventions were included on the basis of their characteristics (e.g. intensity) rather than the name of the approach or model being followed. Studies were eligible for inclusion if they

- included children with a diagnosis of autism based on any editions of the Diagnostic and Statistical Manual of Mental Disorders (*DSM*) (American Psychiatric Association, 2013) or International Classification of Diseases (ICD) criteria (World Health Organization, 2018),
- used ABA-based teaching strategies as the core components of intervention, delivered face-to-face by trained providers for at least 15 h per week, on a one-to-one or small group basis (two or three children per adult),
- used a comprehensive approach, targeting a range of behaviours, skills and developmental domains; studies of narrowly targeted interventions aimed at a single behaviour (e.g. joint attention) were excluded,
- were child-focussed (studies of interventions delivered to parents were excluded),
- were prospective randomised controlled trials or non-randomised controlled studies.

There was no restriction by age, though our primary focus was on children of pre-school age (under 5 years in the United Kingdom). The study comparator for the overall review (Rodgers et al., 2020) could be any non-intensive ABA-based intervention. However, the IPD-MAs and hence, this article focuses on studies that had a TAU or eclectic intervention as comparator. Comparators were classified as ‘eclectic’ when individual children in a study were reported to have received a mix of specified teaching approaches, such as Treatment and Education of Autistic and Related Communication Handicapped Children (TEACCH) (Mesibov & Shea, 2010); Picture Exchange Communication System (PECS) (Carr & Felce, 2007); other behavioural or developmental programmes; speech and language therapy;; music therapy or occupational therapy. Comparators were classified as TAU when individual children in a study were not reported as receiving a particular treatment plan other than what they would normally receive or where the details of the comparator treatment were not provided. Studies comparing high intensity-to-low intensity ABA and different forms of ABA are considered elsewhere (Rodgers et al., 2020). Non-comparative single-arm studies were excluded. There was no restriction by language or date of publication.

### Study identification and data collection

Bibliographic searches of the Cochrane Central Register of Controlled Trials (CENTRAL); CINAHL, Embase, ERIC, MEDLINE, PsycINFO and social science citation index were performed in August 2017 and updated in June 2019. An example of this search strategy is provided in Supplementary File 1. Relevant trial registries were searched to identify ongoing studies. Conference proceedings, dissertations and thesis registries were also searched to identify grey literature. Citations of published studies were examined for further relevant evidence. Finally, authors of identified studies were asked to identify any additional potentially relevant studies.

Titles and abstracts of all identified literature were screened independently by two researchers, as were full publications of potentially relevant trials. Discrepancies were resolved by discussion.

Eligible study investigators were then invited to supply individual-level data, which were harmonised by either the investigators or the research team using standardised coding developed for the project. Data were requested for all recruited children, including any who were excluded from the original published study analyses. All IPDs were checked on receipt by two researchers. Data were checked for internal consistency, and integrity of randomisation (where conducted) and patterns of missing data were examined. Baseline data were tabulated and compared with the study publication and any inconsistencies noted. Data discrepancies were discussed with trial investigators and any errors corrected.

#### Critical appraisal of studies

Risk of bias in RCTs was assessed using the Cochrane Risk of Bias 2.0 tool (RoB 2.0) (Cochrane Methods Group, 2019). Non-randomised controlled study designs were assessed using the ROBINS-I tool (Sterne et al., 2016). The quality of the supplied IPD was also assessed (e.g. whether there was evidence of non-random allocation or substantial missing or incoherent data). This information was used alongside RoB 2.0 and ROBINS-I findings to evaluate the overall quality of the studies. Assessment was undertaken independently by two researchers, with any discrepancies resolved by consensus or recourse to a third researcher if necessary.

### Statistical methods

Outcomes were analysed at 1 and 2 years after recruitment, with additional limited analyses at 3, 4 and 7 years for some domains. Mean differences between early intensive ABA and TAU/eclectic arms were used as the main outcome measure. Analyses using standardised mean differences were performed as a sensitivity analysis for each outcome.

Our main meta-analyses used linear mixed models, which incorporated random effects to allow for heterogeneity across trials and included all data from all trials in a single regression model. Analysis of covariance (ANCOVA) models (Riley et al., 2013) were used, which regress the final outcome values against treatment and baseline values, with random intercept and intervention effects, to account for heterogeneity.

In order to incorporate data captured at multiple time-points, repeated measures analyses were performed. These models analysed all time-points simultaneously, with a single model estimating effects for all reported years.

To explore potential effect modifiers, we investigated the impact of covariates such as age at enrolment, sex, baseline IQ or baseline composite VABS (Vineland adaptive behaviour scale) (Sparrow et al., 1984)) scores on the effectiveness of early intensive ABA-based interventions (intervention–covariate interaction). To do this, the ANCOVA regression models were extended to include a parameter for the covariate of interest and one for the intervention-covariate interaction. Each covariate (except sex) was analysed as a continuous covariate in the regression models. These models were fitted for each possible combination of outcomes and covariates to assess the associations between intervention and covariates, provided sufficient data were available.

Although linear mixed effect ANCOVA models were used for the main analyses, we also performed conventional two-stage random-effects meta-analyses for comparison and to produce forest plots. For these analyses, ANCOVA models were fitted within each trial regressing outcome against treatment, adjusted for baseline levels. Summary mean differences with their standard errors for each trial were then pooled across trials using DerSimonian–Laird random-effects meta-analyses. Heterogeneity was assessed using I^2^ (Higgins & Thompson, 2002).

#### Studies not supplying IPD

For eligible studies that did not supply IPD, two reviewers extracted relevant data from the study publications, such as means and standard deviations for each study arm or mean differences between arms if full data were unavailable. Disagreements were handled through discussion and referred to the primary investigator where appropriate. Mean differences for each outcome measure were calculated from extracted data and then combined with the effect estimates for each study calculated from the IPD, in exploratory random-effects DerSimonian–Laird meta-analyses.

## Results

### Eligible studies

**Figure 2.**
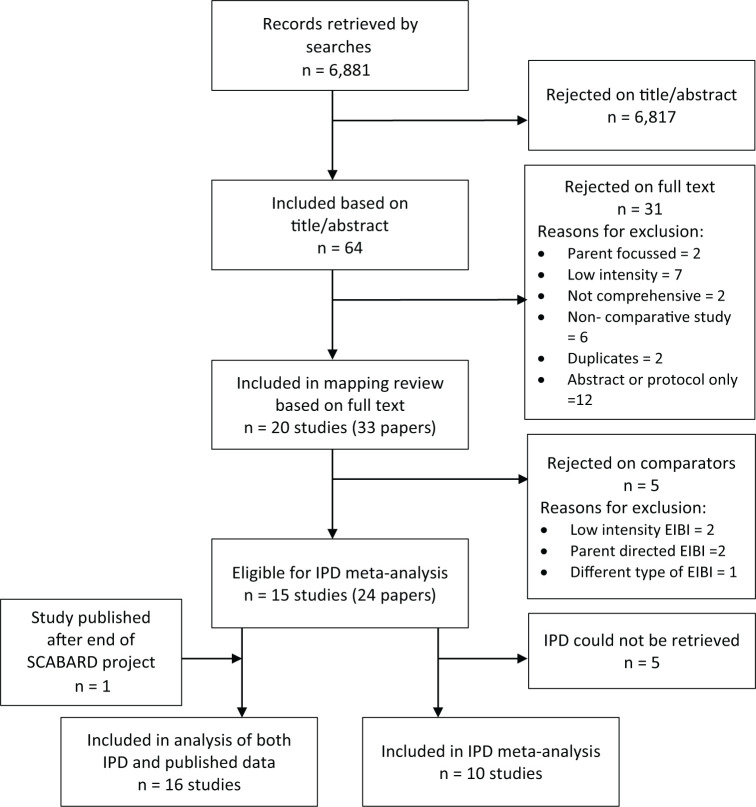
PRISMA flow diagram for full SCABARD review (Rodgers et al., 2020).

After screening the title and abstracts of 6881 records, the full text of 41 studies was examined. Of these, 20 studies met the broader SCABARD inclusion criteria; five further studies were excluded from the IPD-MA because their comparator group did not meet the inclusion criteria for the IPD synthesis (being low intensity, parent-directed or other form of EIBI rather than TAU or eclectic intervention; see Figure 2).

In total, 15 studies (reported in 24 papers published between 1993 and 2017), including 720 participants, were eligible for inclusion in the IPD-MA (Birnbrauer & Leach, 1993; Cohen et al., 2006; Dawson et al., 2010, 2012; Eikeseth et al., 2002, 2007, 2012; Eldevik et al., 2012; Estes et al., 2015; Farrell et al., 2005; Haglund et al., 2017; Howard et al., 2005, 2014; Kovshoff et al., 2011; Magiati et al., 2007; Magiati et al., 2011; Reed et al., 2007; Remington et al., 2007; Vivanti et al., 2014; Zachor & Ben-Itzchak, 2010; Zachor et al., 2007).

After the completion of the SCABARD project, a further study (Rogers et al., 2019) which compared an ABA-based early intensive intervention against an eclectic intervention was published. It was too late to obtain and include IPD at that point, but aggregate data were extracted from the publication and included in analyses that combined IPD and published data (Figure 2). Studies reported across different publications are referenced throughout this article using the earliest peer reviewed article.

### IPD received from eligible studies

We received IPD for 10 of the 15 eligible studies including a total of 491 participants, and accounting for 68% (491/720) of all known (published and unpublished) participant data or 78% (491/626) of the published data. IPD were not available for five studies. For two of these, the authors no longer had access to the data (Birnbrauer & Leach, 1993; Farrell et al., 2005). Two declined to participate (Haglund et al., 2017; Howard et al., 2005), one of which indicated that their data were not yet published (Haglund et al., 2017). IPD from one study (Dawson et al., 2010) could not be separated from a larger data set within the National Database for Autism Research (NDAR). When contacted, the authors replied that they were unable to provide complete IPD due to the study’s original terms of consent.

The mean age of participants at baseline in the 10 available trials was 38.4 months and 87.5% were male. The mean baseline IQ was 59.4% and 74.2% of the participants had an IQ less than 70. The mean baseline VABS composite score was 63.1 (Table 1).

**Table 1.** Baseline characteristics of participants from the IPD data sets.

Study	Group	No. of ptps^a^	Age in months Mean (SD)	Percentage of males	IQ Mean (SD)	VABS composite Mean (SD)	Percentage of ptps with IQ < 70	Percentage of ptps with VABS < 60
Cohen et al. (2006)	Int	21	30.5 (5.5)	85.7	62 (16.4)	69.8 (8.1)	71.4	5
	Com	21	32.4 (3.7)	81	59.4 (14.7)	70.6 (9.6)	76.2	4.8
	Total	42	31.5 (4.7)	83.3	60.7 (15.5)	70.2 (8.8)	73.8	4.9
Eikeseth et al. (2002, 2007)	Int	13	66.3 (11.3)	61.5	61.9 (11.3)	55.8 (9)	69.2	69.2
	Com	12	65.7 (10.4)	91.7	67.3 (16.4)	60 (13.2)	75	41.7
	Total	25	66 (10.6)	76	64.5 (14)	57.8 (11.2)	72	56
Eikeseth et al. (2012)	Int	35	46.8 (11.4)	82.9		67 (10.2)		22.9
	Com	24	53 (14.2)	83.3		63.6 (8.1)		29.2
	Total	59	49.3 (12.9)	83.1		65.6 (9.5)		25.4
Eldevik et al. (2012)	Int	31	42.2 (9.0)	80.6	51.6 (16.9)	62.5 (8.2)	87.1	45.2
	Com	12	46.2 (12.4)	66.7	51.7 (18.1)	58.9 (7.8)	83.3	58.3
	Total	43	43.3 (10.1)	76.7	51.6 (17.0)	61.5 (8.1)	86	48.8
Magiati et al. (2007, 2011)	Int	28	37.9 (7.3)	96.4	49.1 (14.9)	59.6 (6.2)	85.7	50
	Com	16	42.4 (7.6)	75	42.8 (13.1)	55.4 (5.4)	100	71.4
	Total	44	39.5 (7.7)	88.6	46.3 (14.2)	58.1 (6.2)	92	57.5
Reed et al. (2007)	Int	14	42.9 (14.8)	92.9	57.2 (17.8)	59.3 (10.1)	78.6	64.2
	Com1	20	43.7 (4.4)	95	51.9 (20.1)	53 (4.6)	90	95
	Com 2	16	38.1 (8.3)	87.5	53.3 (16.1)	58.6 (6)	87.5	68.8
	Total	50	41.7 (9.6)	92	53.8 (18)	56.5 (7.4)	86	78
Remington et al. (2007) Kovshoff et al. (2011)	Int	23	35.7 (4)	87	61.4 (16.7)	60.2 (5.8)	69.6	39.1
	Com	18	38.9 (3.9)	88.9	63.8 (14.0)	57.2 (7.0)	72.2	72.2
	Total	41	37.1 (4.2)	87.8	62.5 (15.4)	58.9 (6.5)	70.7	53.7
Vivanti et al. (2014)	Int	27	40.3 (9.6)	85.2		68.7 (12.6)		22.2
	Com	30	42 (6.7)	90		68.5 (9.2)		23.2
	Total	50	41.2 (8.2)	87.7		68.6 (10.9)		22.8
Zachor et al. (2007)	Int	26	26.3 (3.8)	97.2	72.3 (16.8)	62.4 (7.8)	47.1	32
	Com	17	28.4 (3.6)	94.1	81.6 (17.8)	63.6 (5.9)	28.6	25
	Total	43	26.9 (3.8)	96.2	75 (17.4)	62.9 (7.0)	41.7	29.3
Zachor & Ben-Itzchak (2010)	Int	49	24.8 (3.9)	91.8		66.4 (6.5)		16.7
	Com	28	25.9 (4.7)	89.3		68.1 (6.2)		11.1
	Total	77	25.2 (4.2)	90.9		67 (6.4)		14.5
Mean across all studies			39 (12.9)	87.2	59.8 (18.4)	63.3 (9.4)	73.4	36.7

Int: intervention; Com: comparator; Ptps: participants; IQ: intelligence quotient; VABS: Vineland adaptive behaviour scale; SD: standard deviation.

a Some studies provided data for more participants and some for fewer than in the original publication. See Rodgers et al. (2020) for full details.

Outcomes were not reported consistently across studies or time-points. A list of all outcome measurement instruments collected is provided in Supplementary File 2, followed by tables indicating which outcomes were collected by each study (Table 4 in Supplementary File 2) and at each time-point (Table 5 in Supplementary File 2). All studies provided IQ, using a variety of measures, and adaptive behaviour IPD using VABS, but data on other outcomes were available for only a subset of studies, particularly for autism symptom severity for which only three studies supplied data (Magiati et al., 2007; Vivanti et al., 2014; Zachor & Ben-Itzchak, 2010). Studies also varied in how language was assessed; three used Reynell developmental language scales (RDLSs) (Reynell & Huntley, 1985; Edwards et al., 1997) and two used Mullen scales of early learning (MSEL) (Mullen, 1995). Most measured outcomes 1 or 2 years after recruitment, but not always both. Data on outcomes beyond 2 years were very limited.

### Description of interventions

Five of the 10 available studies examined variants of the original UCLA EIBI intervention model with use of additional manualised ABA procedures and without the use of aversive techniques (Eikeseth et al., 2002; Eldevik et al., 2012; Reed et al., 2007; Zachor & Ben-Itzchak, 2010; Zachor et al., 2007). The remaining five incorporated some or all aspects of NDBI (Cohen et al., 2006; Eikeseth et al., 2012; Magiati et al., 2007; Remington et al., 2007; Vivanti et al., 2014), with one specifically examining the ESDM approach delivered in a group-format with a child–staff ratio of 1:3 (Vivanti et al., 2014). In all studies, children received early intensive ABA-based interventions for between 12 and 36 months, at a planned intensity of 15–40 h per week.

Eight of the ten studies contained an eclectic comparator arm (Eikeseth et al., 2002, 2012; Eldevik et al., 2012; Magiati et al., 2007; Reed et al., 2007; Vivanti et al., 2014; Zachor & Ben-Itzchak, 2010; Zachor et al., 2007) and all of these comparators were delivered in a school or nursery classroom setting. TAU comparator interventions comprised non-autism specific special education or other forms of standard local provision. Of the three with a TAU comparator, two were delivered in settings outside the home (Cohen et al., 2006; Remington et al., 2007) and one was conducted in the child’s home (Reed et al., 2007). All comparator arms were delivered for a similar duration to experimental arms, though treatment intensity was more variable, ranging from 2 to 40 h per week (where recorded), with considerably less one-to-one contact and mostly delivered in group settings.

### Study quality and risk of bias

All ten studies included in the IPD-MA were non-randomised and rated as being at ‘serious’ risk of bias for at least one domain (Table 2). All studies used convenience samples, with allocation to study arms being pre-determined or based on location or parental preference. Outcome assessors were aware of which intervention the child received in nine studies (Eikeseth et al., 2002, 2012; Eldevik et al., 2012; Magiati et al., 2007; Reed et al., 2007; Remington et al., 2007; Vivanti et al., 2014; Zachor & Ben-Itzchak, 2010; Zachor et al., 2007). We were unable to obtain any study protocols against which to judge adherence to pre-specified methods. These concerns suggest that all results should be interpreted cautiously. It is not possible to quantify how these potential biases influenced the direction and magnitude of the study results. However, given their non-randomised design, the prevalence of parental preference for early intensive ABA-based interventions in some studies and the lack of blind assessors, the effects we have observed in the meta-analysis may be an overestimation of the true effects.

**Table 2.** Risk of bias of studies included in IPD meta-analyses using the ROBINS-I tool.

Study	Confounding	Selection of participants	Classification of interventions	Deviations from intended interventions	Missing data (IPD)	Measurement of outcomes
Cohen et al. (2006)	Serious	Moderate	Low	Moderate	Low	Moderate
Eikeseth et al. (2002)	Serious	Moderate	Low	Moderate	Low	Moderate
Eikeseth et al. (2012)	Serious	Moderate	Low	Moderate	Low	Serious
Eldevik et al. (2012)	Serious	Moderate	Low	Moderate	Low	Serious
Magiati et al. (2007)	Serious	Moderate	Low	Serious	Low	Moderate
Reed et al. (2007)	Serious	Moderate	Low	Moderate	Low	Moderate
Remington et al. (2007)	Serious	Moderate	Low	Moderate	Low	Moderate
Vivanti et al. (2014)	Serious	Serious	Low	Moderate	Low	Serious
Zachor et al. (2007)	Serious	Moderate	Low	Moderate	Low	Serious
Zachor & Ben-Itzchak (2010)	Serious	Moderate	Low	Moderate	Low	Serious

IPD: individual participant data.

### IPD-MAs

Given the small numbers of studies and participants available, the meta-analyses compared any early intensive ABA-based intervention with any TAU/eclectic intervention, without differentiating between intervention types.

#### Adaptive behaviour

**Figure 3.**
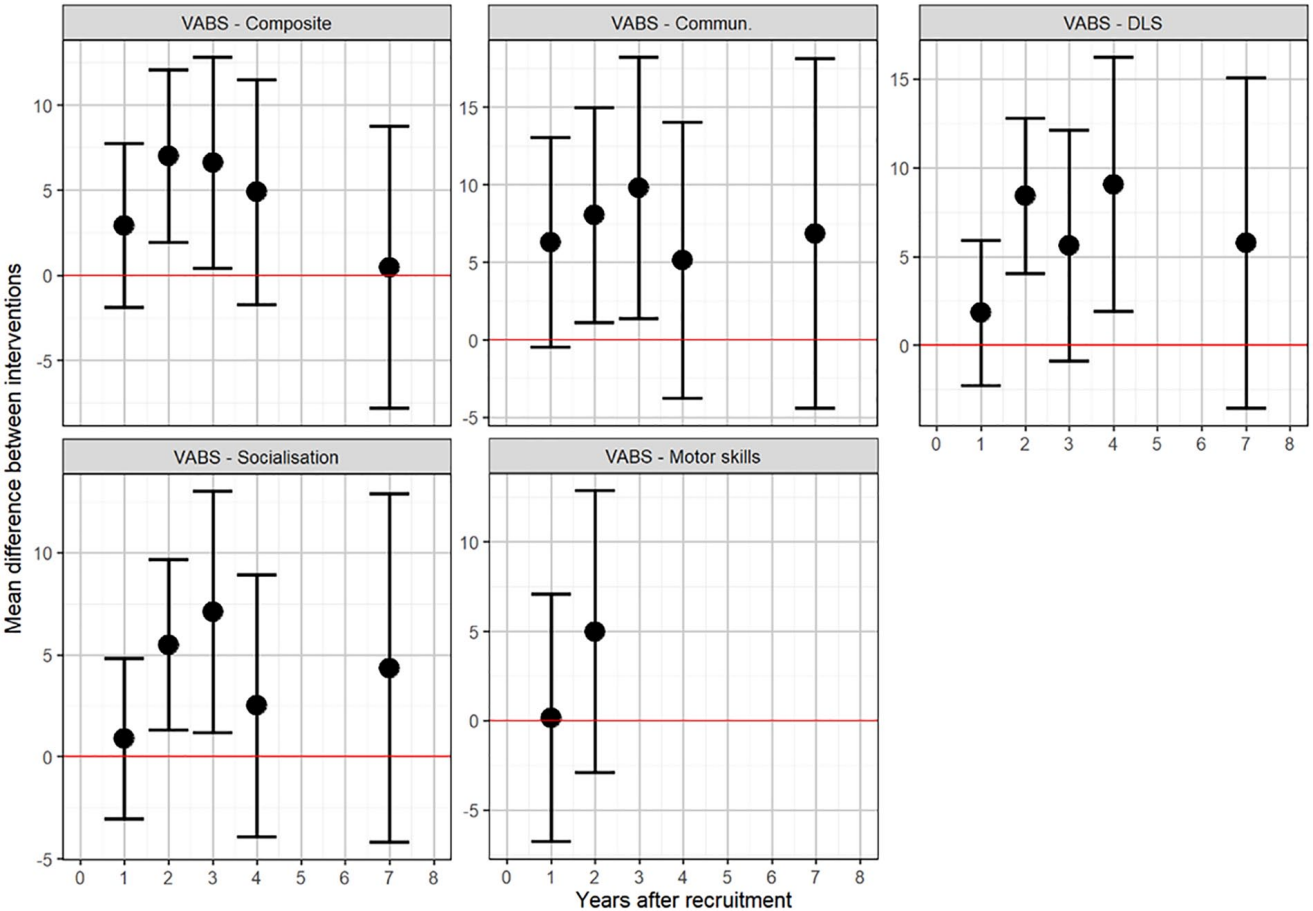
Results of repeated measures meta-analyses of VABS components.

Adaptive behaviour data (as measured using VABS composite score) were provided for all ten of the available trials. Figure 3 shows the results from the repeated measures meta-analyses of adaptive behaviour. The circles show the summary effect estimate for each analysis, with the 95% confidence interval (CI) given by the bars. Each estimate represents an independent meta-analysis for each year; no time trends are assumed. Composite VABS score showed no clear evidence of a difference between experimental and control groups at 1 year (MD = 2.93; 95% CI = –1.90 to 7.76), but a difference of approximately seven points (one-half of a standard deviation (SD)) in favour of early intensive ABA-based intervention after 2 years (MD = 7.00; 95% CI = 1.95–12.06). Results for the individual components of VABS were consistent with the composite score. Longer-term follow-up for VABS was limited to one study (Magiati et al., 2011), with no evidence of any benefit of early intensive ABA at 7 years, although it should be noted that this particular study found no evidence of a benefit of the ABA-based intervention at any follow-up time compared to the eclectic group.

**Figure 4.**
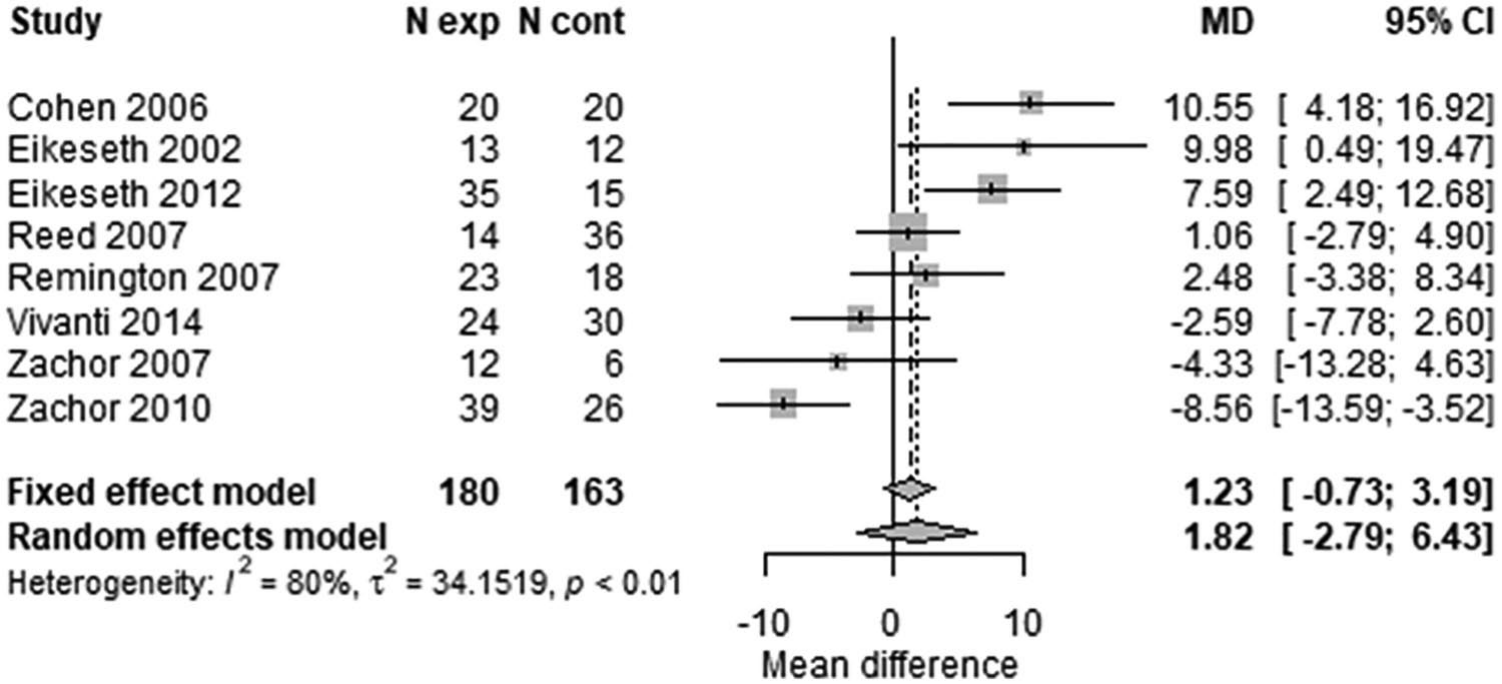
Two-stage random-effects meta-analysis of composite VABS score at 1 year.

**Figure 5.**
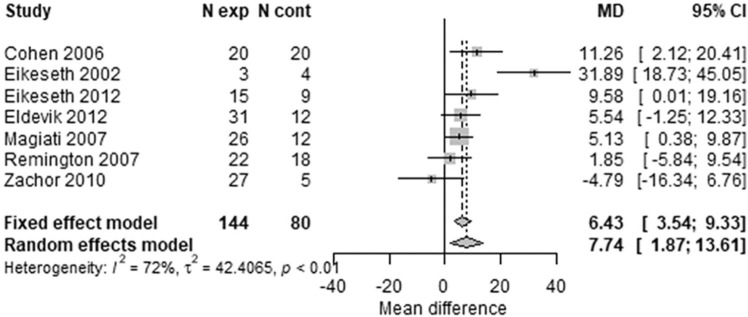
Two-stage random-effects meta -analysis of composite VABS score at 2 years.

An alternative analysis, using conventional meta-analysis methods without allowing for repeated measures, showed similar results, as illustrated by forest plots for VABS (Figures 4 and 5). The VABS composite score including in all 10 available trials, provided no clear evidence of benefit of early intensive ABA-based intervention at 1 year, with substantial heterogeneity (MD = 1.82; 95% CI = –2.79 to 6.43; I^2^ = 80%). However, there was a seven-point difference (one-half of an SD) in favour of early intensive ABA-based intervention after 2 years, with less heterogeneity (MD = 7.74; 95% CI = 1.87–13.61; I^2^ = 34%). Studies varied substantially in their estimated mean differences. One extreme outlier study (Eikeseth et al., 2002), including only seven children after 2 years, found a 32-point difference in favour of early intensive ABA. In the opposite direction, one trial found a five-points difference in favour of the comparator intervention after 2 years (Zachor & Ben-Itzchak, 2010).

#### Cognitive ability (IQ)

IQ was reported in seven of the available studies (Cohen et al., 2006; Eikeseth et al., 2002; Eldevik et al., 2012; Magiati et al., 2007; Reed et al., 2007; Remington et al., 2007; Zachor et al., 2007). A variety of scales were used to measure IQ/cognitive ability. Most studies used the recognised standardised measures (Wechsler pre-school and primary scale of intelligence (WPPSI) (Wechsler, 1989), Wechsler intelligence scale for children (WISC) (Wechsler, 1974) or the Standford–Binet test (SB) (Roid & Pomplun, 2012)) for children of the appropriate age and cognitive level. For children unable to score on these tests due to basal effects, either the Bayley scales of infant development (BSID) (Bayley, 2006) or the Psychoeducational profile–revised (PEP-R) (Schopler et al., 1990) were used. One study (Reed et al., 2007) used only the PEP-R at all time-points (see Table 3). As all these scales were standardised (mean 100 with SD of 15), in the primary analysis of IQ, we have not differentiated between the measures used to assess cognitive ability and assumed equivalence.

**Table 3.** Summary of scales used to measure cognitive ability (IQ) in each study.

Study	Scales used to measure IQ
Cohen et al. (2006)	BSID-R, WPPSI-R
Eikeseth et al. (2002, 2007)	BSID-R, WPPSI-R, WISC-R
Eikeseth et al. (2012)	None
Eldevik et al. (2012)	BSID 2/3, WPPSI-R, SB 4/5
Magiati et al. (2007, 2011)	BSID-R, WPPSI-R, WISC-IV
Reed et al. (2007)	PEP-R
Remington et al. (2007) Kovshoff et al. (2011)	BSID, SB-4
Vivanti et al. (2014)	None
Zachor et al. (2007)	BSID-II, SB-4
Zachor & Ben-Itzchak (2010)	None

IQ: intelligence quotient; BSID: Bayley scales of infant development; WPPSI: Wechsler pre-school and primary scale of intelligence; WISC: Wechsler intelligence scale for children; SB: Standford–Binet test; PEP-R: psychoeducational profile–revised.

**Figure 6.**
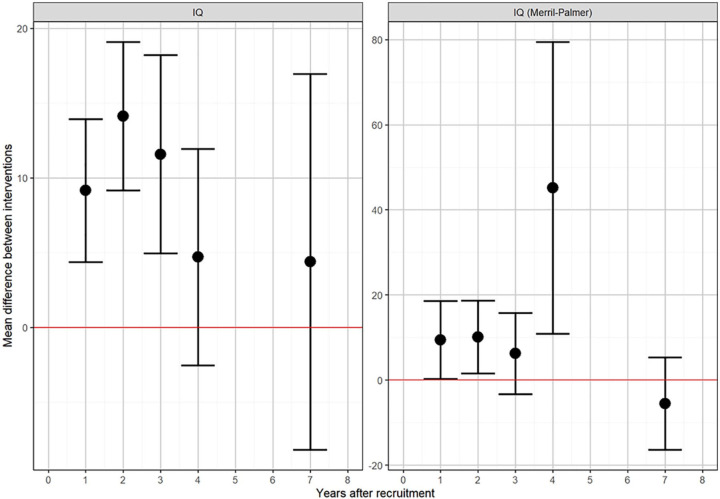
Results of repeated measures meta-analyses of IQ.

Figure 6 shows the results from the main repeated measures meta-analyses which favoured early intensive ABA-based interventions 1 year after follow-up, with a mean difference between groups of around nine points (two-thirds of an SD) in favour of early intensive ABA-based intervention (MD = 9.16; 95% CI = 4.38–13.93). After 2 years of intervention, this increased to a 14-point difference (almost a full SD) in favour of early intensive ABA-based intervention (MD = 14.13; 95% CI = 9.16–19.10). Results after 7 years are based on only one study (Magiati et al., 2007) that found no statistical evidence of a significant difference between the two intervention groups at 7 years (MD = 4.39; 95% CI = –8.17 to 16.95).

The meta-analysis results for non-verbal IQ measured using Merrill–Palmer scale of mental tests (MPSMT; Roid & Sampers, 2004) were based on three studies (Cohen et al., 2006; Eikeseth et al., 2002; Magiati et al., 2007). The results of these analyses were broadly similar to general IQ at both 1 year (MD = 9.45; 95% CI = 0.33–18.59) and 2 years (MD = 10.13; 95% CI = 1.58–18.68), with mean differences between groups of around ten points (two-thirds of an SD) in favour of the early intensive ABA-based interventions after 2 years.

**Figure 7.**
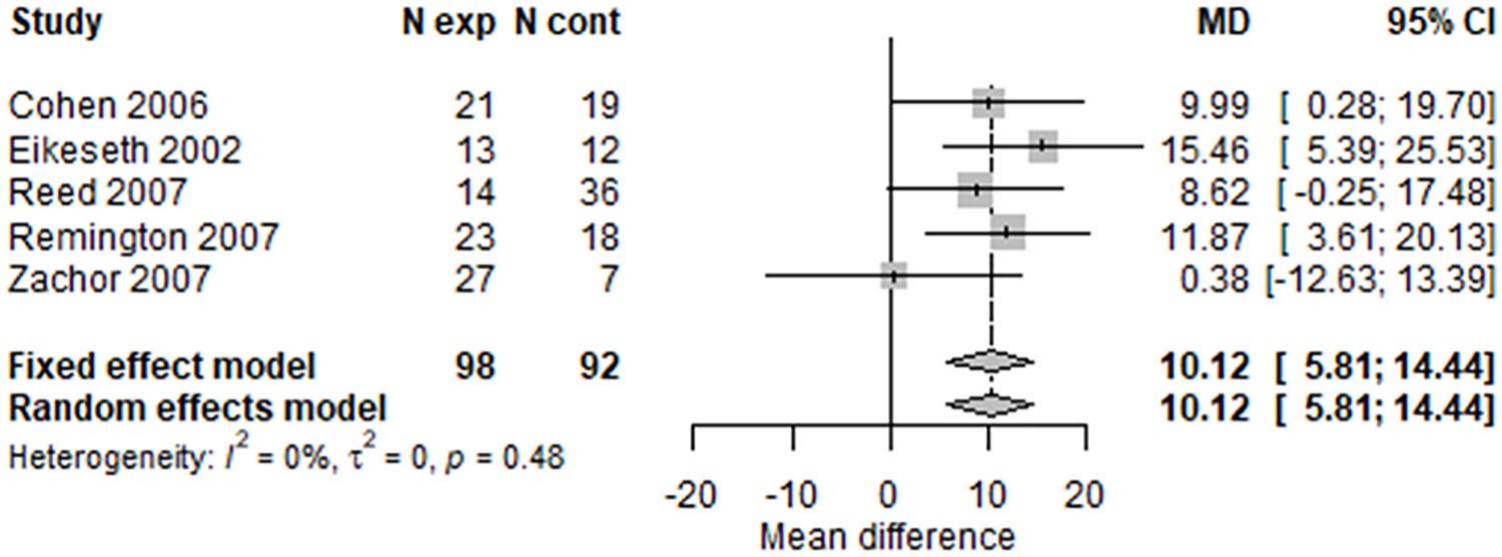
Two-stage random-effects meta-analysis of IQ at 1 year.

**Figure 8.**
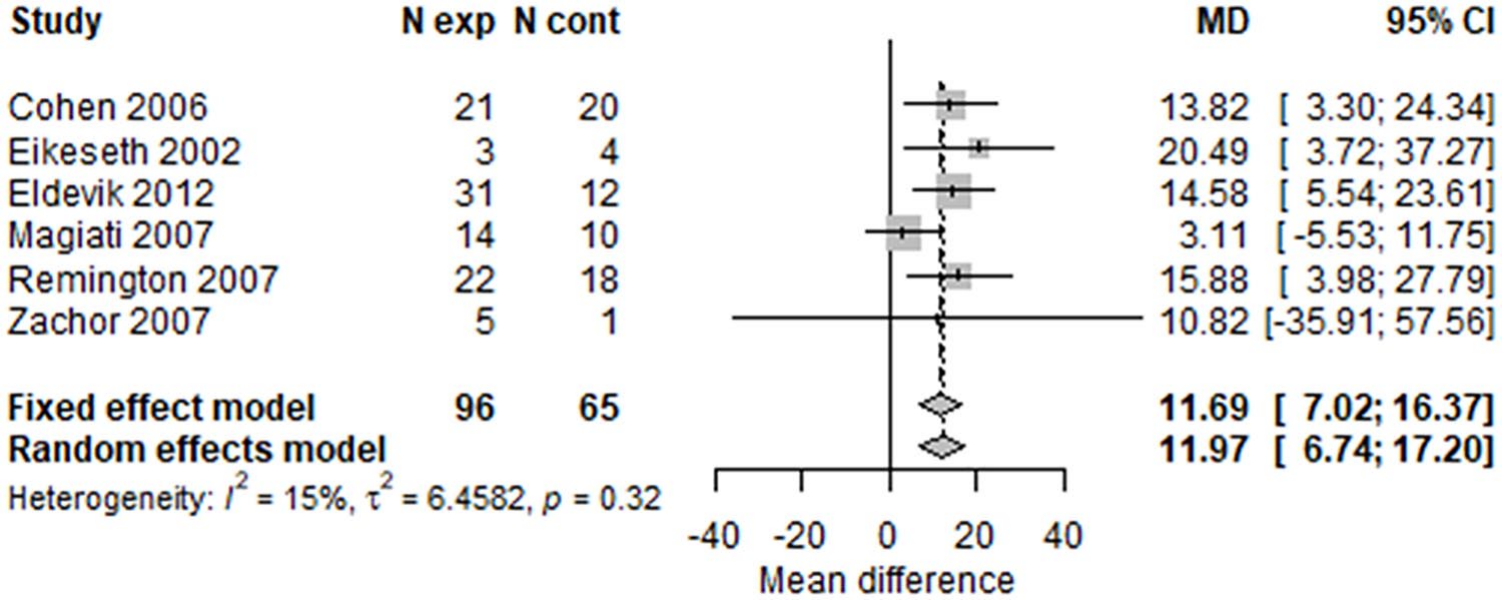
Two-stage random-effects meta-analysis of IQ at 2 years.

Using conventional meta-analysis methods without allowing for repeated measures, the forest plots for IQ (Figures 7 and 8) at 1 and 2 years showed broadly similar results. There was a difference in favour of early intensive ABA of around 10 IQ points (two-thirds of an SD) after 1 year (MD = 10.12; 95% CI = 5.81–14.44; I^2^ = 0); and of 12 IQ points (three-quarters of an SD ) after 2 years (MD = 11.97; 95% CI = 6.74–17.20; I^2^ = 15%).

#### Autism symptom severity

Data for other autism symptom severity and all other outcome domains were extremely limited. Consequently, results were highly uncertain (see Figure 9 in Supplementary File 3).

There was no clear evidence of a significant difference between early intensive ABA-based and TAU/eclectic interventions for the autism diagnostic observation schedule (ADOS) (Lord et al., 2000) calibrated severity scores (Gotham et al., 2007) at 1 or 2 years or for the ADOS repetitive behaviours and social subscales. These analyses were based on only three studies (Magiati et al., 2007; Vivanti et al., 2014; Zachor et al., 2007) and a small number of participants. It was not possible to perform meta-analyses on any other measures of autism symptom severity as none were used in more than a single study.

#### Language

Two different language tools were used: RDLS and MSEL (expressive and receptive language subscales). No studies used both tools. Results were inconsistent between studies using the RDLS (Cohen et al., 2006; Eikeseth et al., 2002; Magiati et al., 2007), which generally showed a benefit of early intensive ABA-based intervention and those using MSEL (Vivanti et al., 2014; Zachor & Ben-Itzchak, 2010), where there was no evidence of a benefit on language. RDLS comprehension scores after 1 year showed a mean difference of about 12 points between arms, favouring early intensive ABA-based interventions (MD = 12.96; 95% CI = 2.01–23.91) and at 2 years (MD = 11.78; 95% CI = 2.12–21.45). Effect estimates were similar for the RDLS expressive language subscale. By contrast, MSEL receptive and expressive language subscales showed no evidence of any difference between early intensive ABA-based and control arms after either 1 or 2 years.

#### Three and four year follow-up analyses

Estimates of effect on all outcome measures at 3 and 4 years are derived from only three studies (Cohen et al., 2006; Eikeseth et al., 2002; Remington et al., 2007), but are generally consistent with effect estimates at other times, having similar estimated mean differences. The exception is a suggested large effect on non-verbal IQ (Merrill–Palmer) after 4 years, but this is based on one study with very few (seven in total) children (Eikeseth et al., 2002).

#### Child characteristics as moderators

Data were insufficient to permit planned investigation of most child-level covariates. We were only able to examine age, sex, baseline IQ and baseline composite VABS scores (see Table 6 in Supplementary File 3). All results are consistent with there being no interaction between these factors and either IQ or VABS score. However, all analyses had very wide confidence intervals indicating a lack of evidence and substantial uncertainty as to whether age, sex, baseline IQ or baseline VABS variables might influence the effectiveness of intervention. For example, there was no clear evidence that the younger children in the data set gained greater benefit from early intensive ABA-based interventions than the older children.

#### Meta-analysis including published data from studies not providing IPD

We performed sensitivity analyses of IQ and composite VABS score at 2 years including data for the five studies that did not provide IPD (Birnbrauer & Leach, 1993; Dawson et al., 2010; Farrell et al., 2005; Haglund et al., 2017; Howard et al., 2005) and the sixth which was published after data collection (Rogers et al., 2019).

The baseline characteristics, interventions and comparator groups for these six studies followed a broadly similar pattern to the studies for which IPD were available (see Table 8 in Supplementary File 4). Four of these six studies were non-randomised (Birnbrauer & Leach, 1993; Farrell et al., 2005; Haglund et al., 2017; Howard et al., 2005) and were assessed as being at ‘serious’ bias in at least one domain. The remaining two studies (Dawson et al., 2010; Rogers et al., 2019) were RCTs. Both of these were deemed to have ‘some concerns’ about risk of bias in at least two domains and one was rated at a ‘high’ risk of bias for their follow-up paper (Estes et al., 2015) due to missing outcome data (see Tables 8 and 9 in Supplementary File 4).

The analyses on all included studies including these six produced larger suggested benefits of early intensive ABA-based intervention than the main analyses using only IPD (see Figures 10 and 11 in Supplementary File 4). This appears to be driven by the very large comparative effects found by one study on IQ and VABS (Howard et al., 2005), which were approximately double those estimated from the IPD-MA. The other five studies that did not provide IPD (Birnbrauer & Leach, 1993; Dawson et al., 2010; Farrell et al., 2005; Haglund et al., 2017; Rogers et al., 2019) reported findings on IQ and VABS that were more consistent with the IPD-MAs.

## Discussion

The IPD-MA included 491 individuals from 10 eligible studies that provided data amounting to 68% of all known study participants. The two most recorded outcomes were adaptive behaviour (VABS) and cognitive ability (IQ). Compared with ‘eclectic’ intervention or TAU, early intensive ABA-based interventions had minimal or no significant advantage on VABS standard scores after 1 year compared to TAU/eclectic interventions but showed an average seven-point difference (half an SD) after 2 years. For IQ, an average comparative improvement of approximately 9 points in favour of the EIBI interventions was observed at 1 year and 14 points at 2 years. Data for other outcomes were not consistently collected and too sparse to enable us to conduct a meta-analysis. There was no clear evidence that the interventions were any more or less effective according to the sex or age of a child or IQ or VABs score at baseline.

Sensitivity analyses including summary data extracted from publications for the five studies which did not provide IPD, and for a 60th-study which was published after the SCABARD project concluded, produced larger suggested benefits of early intensive ABA-based intervention than observed in the main analyses using only IPD. However, these larger benefits were mostly driven by a single study (Howard et al., 2005) which reported effects approximately double those estimated from the IPD-MA.

This article reports the findings from the main IPD-MAs on the effectiveness of early intensive ABA-based interventions compared with TAU or eclectic interventions. A potential limitation of this approach is that it might obscure treatment effects from different models considered as procedurally or theoretically distinct. However, as described in the full report, examination of the different treatment effects by theoretical model found no evidence for analysing models separately (Rodgers et al., 2020). This finding, combined with advice from our advisory group and a wider stakeholder consultation, suggests no benefits from such an approach unless better data are available.

All the included studies were at risk of bias. Most were not randomised, with intervention assignment often based on parental preference and outcome assessments were rarely conducted blind. No mechanisms that might safeguard against bias, such as prospective registration and/or publication of study protocols, were undertaken, although many studies predate the era when registration and publication of protocols became established practice. It is notable that a recently published randomised trial (Rogers et al., 2019), replicating the ESDM evaluation by Dawson et al. (2010), which was included in our sensitivity analysis, sought to address some of the concerns about risk of bias in earlier studies. The results of this trial were notably less favourable than our IPD results for the early intensive ABA-based intervention. A systematic review of aggregate data, published during the completion of SCABARD, noticed a similar pattern of results (Sandbank et al., 2020). This review of all interventions for young children with autism reported positive summary effects for several approaches, but when analysis was limited to RCT designs and to outcomes without a risk of detection bias, none showed significant effects on any outcome.

While our results suggest relative benefits in child cognitive ability and adaptive behaviour for participants in early intensive ABA-based interventions relative to TAU/eclectic interventions, only limited conclusions can be drawn because we cannot rule out the possibility that the observed effects in our IPD synthesis could be partly or entirely attributable to bias within the included studies or the quality of the data collected. Apart from the VABs measure, outcomes were not collected consistently, and domains, such as autism symptom severity, behaviours that challenge and education placement, were infrequently collected.

Studies rarely collected data on quality of life, emotional and mental health and well-being of the children and the families or any other socially valid and important outcomes for autistic people and their families as recommended by a review into the use of tools to measure outcomes in autistic children (McConachie et al., 2015). This lack of information about the possible long-term consequences of early intensive ABA-based interventions on subsequent mental health, quality of life and well-being has been previously highlighted (Kupferstein, 2018).

Caution should be taken when interpreting the findings in relation to cognitive ability (IQ), whose data was not as robust as the data for the VABS outcome. As stated in the results, the tools utilised to measure this outcome domain varied, both across and within studies. Although we decided to combine the measures, as if they were assumed to be equivalent, there are some theoretical and practical differences between the tools which put into question the validity of this assumption. For example, the Wechsler and Standford–Binet scales provide a cognitive ability quotient expected theoretically to stay relatively stable over time, whereas the PEP-R and BSID tools list a number of competencies that accrue with age and are usually assessed only in young children. There was also a concern over the validity of the data as the criteria for which test should be used with each individual child differed across studies and also there was a considerable amount of loss to follow-up across studies. Furthermore, there are concerns over the appropriateness and relevance of IQ as a meaningful intervention outcome for autistic children (Crowe & Salt, 2015; Le Couteur & Szatmari, 2015), something which was also voiced by many of members of the advisory group.

Many of the participant, family, and treatment variables we intended to evaluate were generally not collected or reported in the studies. Other potential variables of interest that we had not specified (e.g. ethnicity and socioeconomic status) were also largely absent. Absence of such data also meant that we were unable to explore whether treatment effect was different for particular sub-groups of children. Longer-term outcome data were notably missing, with most of the studies measuring outcomes up to only two years after recruitment. The one study with longer-term follow-up showed no evidence of significant relative benefits of EIBI versus eclectic interventions at any time-point up to seven years (Magiati et al., 2011).

Of further note, there were almost no data on possible adverse effects of intervention and comparator therapies. Concerns have also been raised about whether EIBI discourages spontaneity and interactive communication, restricts the child’s capacity to develop generalisation skills and increases the risk of behaviours that challenge (Schreibman et al., 2015; Shyman, 2016). While the VABS does collect some information on spontaneous communication and socialisation, the studies did not systematically collect data on adverse events or potential consequences of interventions or comparators. Therefore, in the absence of any systematically collected data, the nature and extent of any potential risks, adverse effects or harms of either early intensive ABA-based intervention or the comparator interventions for the participants, as well as their families in the short, medium of longer term cannot currently be determined.

The studies were conducted over a period of more than twenty years during which time the understanding of research study design as well as of diagnosis and support for autism has evolved. This is of particular concern for studies using TAU as a comparator; older studies may have observed larger effects due to the limited alternative treatment available at the time. Consequently, there is noticeable variation between individual studies in terms of the delivery of interventions and comparators, the conceptualisation of autism and the outcomes of interest. Important contextual information, such as local inclusive educational policies, was also rarely available. Thus, there are likely to be important differences between this body of evidence and the context in which early intensive ABA-based interventions and other treatment alternatives are delivered currently and in the future.

### Suggested research priorities

This review and IPD-MAs comprise the most comprehensive and detailed independent investigation of early intensive ABA-based interventions compared to other eclectic/TAU interventions to date. They were undertaken on behalf of an international collaboration of investigators (including original study authors) and an expert international advisory group (including representation from autistic people, parents and practitioners).

The review involved exhaustive examination of the data at the level of individual children, finding a lack of high-quality evidence to support the effectiveness of early intensive ABA-based interventions compared to TAU/eclectic early interventions. Without obtaining IPD from the five studies that did not collaborate with SCABARD, additional systematic reviews or meta-analyses of studies published to date cannot add any further knowledge, and so are unnecessary. Careful consideration should be given as to whether further primary evaluations of early intensive ABA-based therapy against TAU/eclectic approaches is an appropriate next step, given both the findings from the most recent RCT of effectiveness (Rogers et al., 2019) and the availability of a range of other pre-school autism interventions that fall outside the scope of this review such as social communication interventions delivered through parents or teachers (Kasari et al., 2006; Pickles et al., 2016). However, these interventions also show modest effects and little examination of the longer-term impacts to date.

Therefore, the relative effectiveness of different early intervention approaches remains unclear and there are limitations to the quality of the research evaluation studies conducted to date. Furthermore, as autism is a heterogeneous condition, future research will need to investigate which early interventions, components of early interventions, or combinations of supports or interventions are more effective for children and families. Focusing on mechanisms of action, components of interventions, individual developmental trajectories and wider family and social contextual factors, rather than just on whether a particular named approach or treatment is more or less effective, may well aid the development of new optimised interventions to move the field forward (Green, 2015).

Future clinical trials of early intensive interventions in autism including ABA-based interventions should be conducted by research groups using pre-specified intervention evaluation protocols including an RCT design and agreed core sets of outcome measures collected by trained researchers blind to intervention received. Collecting data on fidelity to treatment received (in both arms), withdrawals and potential adverse events and harms will also be important. Careful characterisation of children and their families and the use of a core set of outcome measures that are meaningful for the autism community will facilitate sharing of findings across clinical trials (McConachie et al., 2018).

Currently, for most early interventions in autism, little is known about the timeframes over which both benefits and harms may become apparent. Retrospective follow-up studies that lack comparative data have reported some contradictory findings with the long-term outcomes of EIBI, with some studies reporting benefits (Smith et al., 2019) and some reporting small but significant decreases in IQ over time (Perry et al., 2017). There are also financial and pragmatic constraints on the collection of long follow-up information; other types of research may need to be employed to address uncertainties. This might include planned follow-up into adolescence and adulthood of children recruited to existing effectiveness studies, retrospective case-control analyses looking at outcomes of children who had followed any early intervention, and/or case-control studies to investigate rates of mental health issues in autistic individuals who have received different interventions in early childhood.

## Conclusion

These IPD-MAs have shown that early intensive ABA-based intervention may lead to larger improvements in child cognitive ability and adaptive behaviour after two years for some children, as compared to TAU/ eclectic interventions. However, all identified studies were at risk of bias, limiting the conclusions that can be drawn, while individual study results varied considerably, with some showing no relative benefit of early intensive ABA-based interventions compared with eclectic/TAU. Furthermore, in common with the evaluation of most autism intervention evaluation studies, there is a lack of reliable longer-term comparative follow-up data. Consequently, there is no clear evidence of whether: (i) any comparative benefits of intervention are retained through and after childhood; (ii) the intervention alters the course of a child’s education; or (iii) it has any comparative benefits on important and meaningful (to the autistic community) outcomes in adulthood including in educational provisions and access, independence, behaviours that challenge and well-being. Using the limited data available, none of the tested individual participant characteristics (sex, baseline age, IQ and composite VABS) moderated the size of the treatment effect, meaning there is no strong evidence to date to identify specific sub-groups of children who might benefit more or less from early intensive ABA-based or eclectic interventions. Furthermore, very few studies have consistently examined more meaningful and important functional outcomes nor intervention characteristics or family/ social environmental influences on intervention outcomes. All these factors are important to explore when considering individual differences in outcomes.

## Supplemental Material

sj-docx-1-aut-10.1177_1362361320985680 – Supplemental material for Intensive behavioural interventions based on applied behaviour analysis for young children with autism: An international collaborative individual participant data meta-analysis Click here for additional data file. Supplemental material, sj-docx-1-aut-10.1177_1362361320985680 for Intensive behavioural interventions based on applied behaviour analysis for young children with autism: An international collaborative individual participant data meta-analysis by Mark Rodgers, Mark Simmonds, David Marshall, Robert Hodgson, Lesley A Stewart, Dheeraj Rai, Kath Wright, Esther Ben-Itzchak, Svein Eikeseth, Sigmund Eldevik, Hanna Kovshoff, Iliana Magiati, Lisa A Osborne, Phil Reed, Giacomo Vivanti, Ditza Zachor and Ann Le Couteur in Autism
