# Supplementary material for: Intensive behavioural interventions based on applied behaviour analysis for young children with autism: An international collaborative individual participant data meta-analysis
Source: Autism. 2021 Jan 22;25(4):1137–53. doi: 10.1177/1362361320985680 (PMC8108110; doi:10.1177/1362361320985680)
Supplement: sj-docx-1-aut-10.1177_1362361320985680 – Supplemental material for Intensive behavioural interventions based on applied behaviour analysis for young children with autism: An international collaborative individual participant data meta-analysis [file sj-docx-1-aut-10.1177_1362361320985680.docx]

**Supplementary File 1: Example search strategy**

**Databases searched:**

CENTRAL, CINAHL, Conference Papers Citation Index, Embase, ERIC, Ethos (British Library Dissertations database), MEDLINE, PsycINFO, Social Sciences Citation Index

Also search two trials registers: ClinicalTrials.gov and WHO ICTRP

A total of 10,843 records identified. After importing into EndNote bibliographic software and deduplicating, there were a total of 6,698 records.

**CENTRAL**

**Via Wiley’s The Cochrane Library**

**Search date 3^rd^ August 2017**

**193 records retrieved**

**Search Name: ABA autism**

**Last Saved: 03/08/2017 14:45:13.322**

#1 MeSH descriptor: [Autistic Disorder] explode all trees

#2 MeSH descriptor: [Autism Spectrum Disorder] explode all trees

#3 MeSH descriptor: [Asperger Syndrome] explode all trees

#4 autism or autistic or ASD or ASDs or ASC or AAC:ti,ab,kw (Word variations have been searched)

#5 asperger*:ti,ab,kw (Word variations have been searched)

#6 #1 or #2 or #3 or #4 or #5

#7 MeSH descriptor: [Applied Behavior Analysis] explode all trees

#8 MeSH descriptor: [Early Intervention (Education)] explode all trees

#9 MeSH descriptor: [Behavior Therapy] explode all trees

#10 " early intervention therapy":ti,ab,kw or " early intervention therapies":ti,ab,kw (Word variations have been searched)

#11 intensive* near/2 (analys* or behavior* or behaviour* or intervention* or program* or therap* or treat*):ti,ab,kw or intensity near/2 (analys* or behavior* or behaviour* or intervention* or program* or therap* or treat*):ti,ab,kw or high-intensity near/2 (analys* or behavior* or behaviour* or intervention* or model* or program* or therap* or treat*):ti,ab,kw or low-intensity near/2 (analys* or behavior* or behaviour* or intervention* or model* or program* or therap* or treat*):ti,ab,kw (Word variations have been searched)

#12 "high intensity" near/2 (analys* or behavior* or behaviour* or intervention* or model* or program* or therap* or treat*):ti,ab,kw or "low intensity" near/2 (analys* or behavior* or behaviour* or intervention* or model* or program* or therap* or treat*):ti,ab,kw or "intensive behavior*" near/2 (analys* or intervention* or model* or program* or therap* or treat*):ti,ab,kw or "intensive behaviour*" near/2 (analys* or intervention* or model* or program* or therap* or treat*):ti,ab,kw (Word variations have been searched)

#13 "early behavior*" near/2 (analys* or intervention* or model* or program* or therap* or treat*):ti,ab,kw or "early behaviour*" near/2 (analys* or intervention* or model* or program* or therap* or treat*):ti,ab,kw or "comprehensive behavior*" near/2 (analys* or intervention* or model* or program* or therap* or treat*):ti,ab,kw or "comprehensive behaviour*" near/2 (analys* or intervention* or model* or program* or therap* or treat*):ti,ab,kw (Word variations have been searched)

#14 "applied behavior*" near/2 (analy* or intervention* or model* or program* or therap* or treat*):ti,ab,kw or "applied behaviour*" near/2 (analy* or intervention* or model* or program* or therap* or treat*):ti,ab,kw or ABA* near/2 (analy* or intervention* or model* or program* or therap* or treat*):ti,ab,kw (Word variations have been searched)

#15 NDBI*:ti,ab,kw or "Naturalistic Developmental Behav* Intervention*":ti,ab,kw or IBI or EIBI or ABA:ti,ab,kw or Lovaas*:ti,ab,kw (Word variations have been searched)

#16 "Early Start Denver Model":ti,ab,kw or "Denver Model":ti,ab,kw or ESDM or ESDM-I or ESDM-PD or P-ESDM:ti,ab,kw (Word variations have been searched)

#17 "Pivotal Response" near/2 (treat* or train* or program*):ti,ab,kw or "PRT train*" or "PRT program*" or "PRT model*":ti,ab,kw or "discrete trial train*":ti,ab,kw (Word variations have been searched)

#18 #7 or #8 or #9 or #10 or #11 or #12 or #13 or #14 or #15 or #16 or #17

#19 #6 and #18

**Supplementary File 2: Outcome Measures**

- Adaptive behaviour
  - Composite Vineland Adaptive Behavioural Scale (VABS) (Sparrow et al. 1984)
  - Each component of the VABS composite score:
    - Communication
    - Daily living skills
    - Socialization
    - Motor skills
    - Maladaptive behaviour (not always recorded)
- Cognitive ability (IQ)
  - Based on specified test:
    - Bayley Scales of Infant Development (BSID I, II or III) (Bayley 1969, 1993, 2006)
    - Psychoeducational Profile-Revised (Schopler et al. 1990)
    - Wechsler Intelligence Scale for Children-Revised (WISC-R) (Wechsler 1949, 1974, 1993)
    - Wechsler Preschool and Primary Scale of Intelligence-Revised (WPPSI-R) (Wechsler 1989, 2002, 2012)
    - Stanford-Binet Test(Roid and Pomplun 2012)
- Non-verbal IQ
  - Merrill-Palmer Scale of Mental Tests (MPSMT) (Roid and Sampers 2004)
- Language development
  - Expressive, receptive, comprehension and overall using scales:
    - Expressive One Word Vocabulary Test (EOWPVT) (Gardner 1990; Brownell 2000)
    - British Picture Vocabulary Scale (BPVS-II) (Dunn et al. 1997)
    - Reynell Developmental Language Scales (RDLS, RDLS-3) (Edwards et al. 1997; Reynell and Huntley 1985)
    - Mullen Scales of Early Learning (MSEL, expressive and receptive language sub-scales)(Mullen 1995)
    - Social Communication Questionnaire (SCQ) (C. Lord and Rutter 2003)
- Autism symptom severity
  - Autism Diagnostic Observation Schedule (ADOS) (Catherine Lord et al. 2000)
  - Autism Diagnostic Interview Revised (ADI-R) (Rutter et al. 2003)
- Presence of behaviours that challenge
  - Conners’ Rating Scales-Revised (CRS-R) (Conners 2001)
- Additional outcomes
  - Other components of MSEL:
    - Composite score
    - Fine motor
    - Visual reception

**Table 4 Outcome measures recorded by study**

| **Outcome type** | **Outcome** | **Cohen 2006** | **Eikeseth 2002/ 2007** | **Eldevik 2012** | **Magiati 2007 /2011** | **Vivanti 2014** | **Eikeseth 2012** | **Reed 2007a/ 2007b** | **Remington 2007 Kovshoff 2011** | **Zachor 2007** | **Zachor 2010** |
| --- | --- | --- | --- | --- | --- | --- | --- | --- | --- | --- | --- |
| Autism severity | ADOS - Rep. behav. | No | No | No | No | Yes | No | No | No | No | Yes |
|  | ADOS Severity score | No | No | No | No | Yes | No | No | No | No | Yes |
| IQ | IQ (any scale) | Yes | Yes | Yes | Yes | No | No | Yes | Yes | Yes | No |
|  | IQ (BSID scale) | No | Yes | No | Yes | No | No | No | No | No | No |
|  | Non-verbal IQ (Merril-Palmer) | Yes | Yes | No | Yes | No | No | No | No | No | No |
| Language | MSEL - Expressive | No | No | No | No | Yes | No | No | No | No | Yes |
|  | MSEL - Receptive | No | No | No | No | Yes | No | No | No | No | Yes |
|  | RDLS - Comprehension | Yes | Yes | No | Yes | No | No | No | No | No | No |
|  | RDLS - Expressive | Yes | Yes | No | Yes | No | No | No | No | No | No |
| Adaptive behaviour | VABS - Commun. | Yes | Yes | Yes | Yes | Yes | Yes | No | Yes | Yes | Yes |
|  | VABS - Composite | Yes | Yes | Yes | Yes | Yes | Yes | Yes | Yes | Yes | Yes |
|  | VABS - DLS | Yes | Yes | Yes | Yes | Yes | Yes | No | Yes | Yes | Yes |
|  | VABS - Maladaptive | No | Yes | No | No | No | Yes | No | No | No | No |
|  | VABS - Motor skills | No | Yes | Yes | Yes | Yes | Yes | No | Yes | Yes | Yes |
|  | VABS - Socialisation | Yes | Yes | Yes | Yes | Yes | Yes | No | Yes | Yes | Yes |
| School placement | School placement | No | No | No | Yes | No | No | No | No | No | No |
| Other | MSEL - Composite | No | No | No | No | Yes | No | No | No | No | Yes |
|  | MSEL - Fine motor | No | No | No | No | Yes | No | No | No | No | Yes |
|  | MSEL - Visual recep. | No | No | No | No | Yes | No | No | No | No | Yes |

**Table 5 Number of studies reporting outcomes at baseline, and 1, 2 and 7 year follow-up timepoints**

| **Outcome** | **Time of reporting** | | | |
| --- | --- | --- | --- | --- |
|  | **Baseline**  **(10 studies)** | **1 year**  **(8 studies)** | **2 years**  **(8 studies) *** | **7 years**  **(1 study)** |
| ADOS - Rep. behav. | 2 | 2 | 1 | 0 |
| ADOS Severity score | 2 | 2 | 1 | 0 |
| IQ (BSID scale) | 2 | 1 | 2 | 1 |
| IQ (Merril-Palmer) | 3 | 2 | 3 | 1 |
| IQ (any scale) | 7 | 5 | 6 | 1 |
| MSEL – Composite | 2 | 2 | 1 | 0 |
| MSEL – Expressive | 2 | 2 | 1 | 0 |
| MSEL– Fine motor | 2 | 2 | 1 | 0 |
| MSEL– Receptive | 2 | 2 | 1 | 0 |
| MSEL– Visual recep. | 2 | 2 | 1 | 0 |
| RDLS – Comprehension | 3 | 2 | 3 | 1 |
| RDLS – Expressive | 3 | 2 | 3 | 1 |
| VABS – Commun. | 9 | 7 | 8 | 1 |
| VABS – Composite | 10 | 8 | 8 | 1 |
| VABS – DLS | 9 | 7 | 8 | 1 |
| VABS – Maladaptive | 2 | 2 | 1 | 1 |
| VABS – Motor skills | 8 | 6 | 7 | 1 |
| VABS – Socialisation | 9 | 7 | 8 | 1 |

* In one study data at two years was available only for a subset of children (Eikeseth et al. 2002, 2007; Wechsler 1949, 1993, 1974)

**Supplementary File 3: Analyses of other outcomes and child-level covariates**


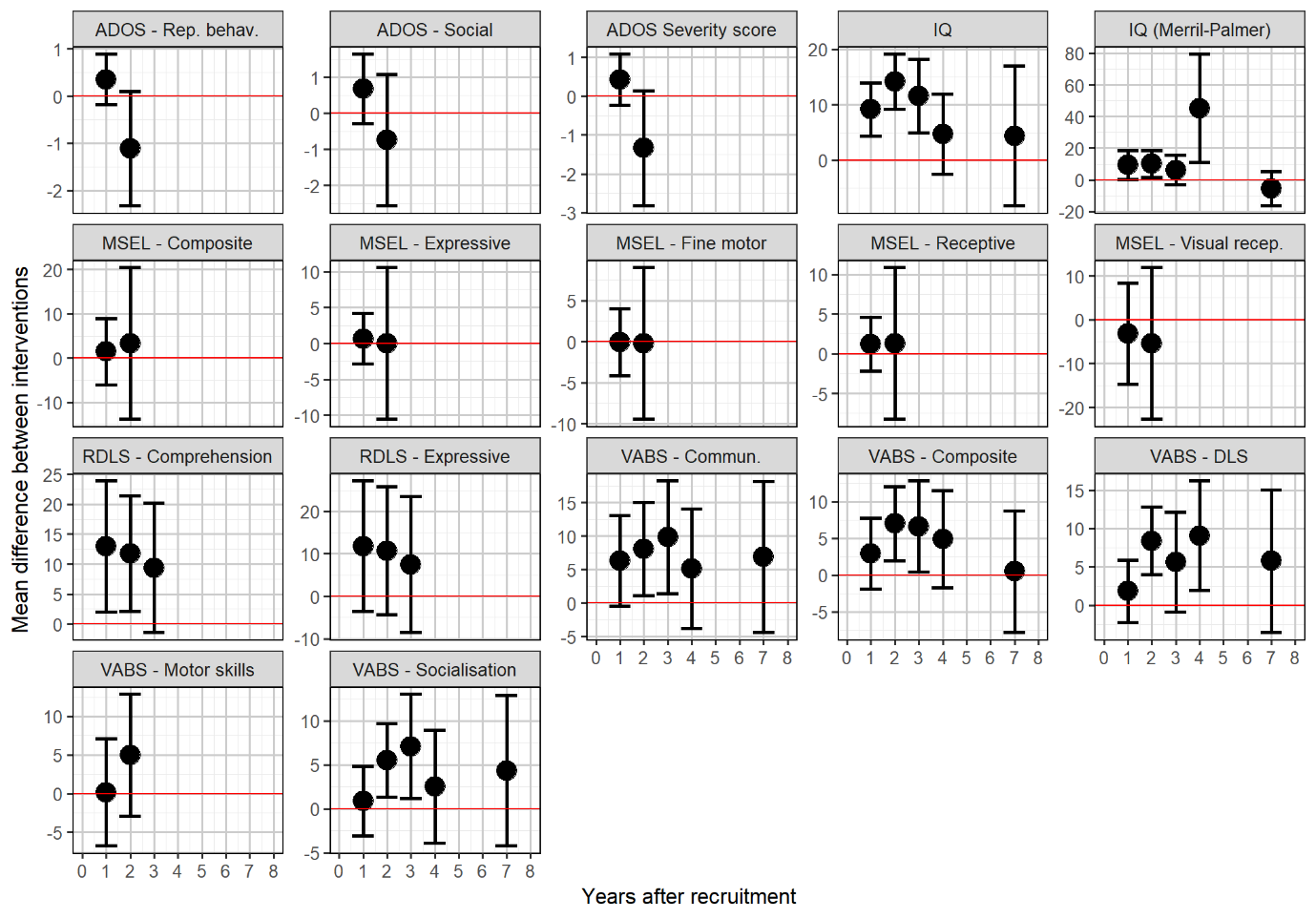


**Fig. 9 Repeated measures meta-analysis of all outcomes**

* Reduction in ADOS score is considered a positive result; for all other scales increased scores are considered positive

**Table 6 Results of child-level interaction models**

| **Outcome** | **Interacting factor** | **Variation in mean difference** | **95% CI** | | **p-value** |
| --- | --- | --- | --- | --- | --- |
|  |  |  |  |  |  |
| IQ | Age at recruitment | 0.08 | -0.26 | 0.42 | 0.64 |
|  | Sex | -1.3 | -13.36 | 10.76 | 0.83 |
|  | IQ at baseline | 0.09 | -0.13 | 0.31 | 0.44 |
|  | VABS at baseline | -0.11 | -0.58 | 0.35 | 0.63 |
|  |  |  |  |  |  |
| VABS composite | Age at recruitment | -0.05 | -0.29 | 0.18 | 0.65 |
|  | Sex | 3.28 | -3.08 | 9.65 | 0.31 |
|  | IQ at baseline | 0.09 | -0.06 | 0.23 | 0.24 |
|  | VABS at baseline | -0.05 | -0.28 | 0.18 | 0.66 |

**Supplementary File 4: Details of Meta-analyses incorporating published data not available as IPD**

This supplement incorporates published data from the five studies that did not provide IPD during the course of the SCABARD project (Birnbrauer and Leach 1993; Farrell et al. 2005; Dawson et al. 2010; Haglund et al. 2017; Howard et al. 2005), and one study (Rogers et al. 2019)that was published after completion of the planned project analyses (Table 7).

**Table 7 Baseline characteristics of included studies**

| **Study** | **Group** | **# of ptps** | **Age in months**  **Mean (sd)** | **% of Males** | **IQ**  **Mean (sd)** | **VABS composite**  **Mean (sd)** |
| --- | --- | --- | --- | --- | --- | --- |
| Birnbrauer 1993 | Int | 9 | 39 (NR) | 44.4 | NR** | NR** |
|  | Com | 5 | 33 (NR) | 100 | NR** | NR** |
| Dawson 2010/2012  Estes 2015  Sullivan 2014 | Int | 24 | 23.9 (4) | NR | 61 (9.2) | 69.5 (5.7) |
|  | Com | 24 | 23.1 (3.9) | 75 | 59.4 (8.6) | 69.9 (7.3) |
| Farrell 2005 | Int | 8 | NR | 75 | NR** | NR |
|  | Com | 9 | NR | 77.8 | NR** | NR |
| Haglund 2017 | Int | 67 | NR | NR | NR | NR |
|  | Com | 27 | NR | NR | NR | NR |
| Howard 2005/2014 | Int | 29 | 30.86 (5.16) | 86.2 | 58.54 (18.15) | 70.46 (11.85) |
|  | Com 1 | 16 | 37.44 (5.68) | 81.3 | 53.69 (13.5) | 69.81 (10.48) |
|  | Com 2 | 16 | 34.56 (6.53) | 100 | 59.88 (14.85) | 71.62 (10.47) |
| Rogers 2019 | Int | 55 | 20.6 (3.37) | 65.1 | 66.98 (18.61) | NR** |
|  | Com | 69 | 20.7 (3.21) | 81 | 64.92 (15.56) | NR** |

Int=Intervention, Com=Comparator, NR=Not Reported

*Baseline data is only presented in the original article for participants who completed treatment. The original sample size was 19.

**These values were reported in the articles as age equivalents rather than means.

**Description of Interventions**

Of the six studies, the interventions provided by two studies closely resembled the original UCLA-developed method of EIBI, though without physical aversive techniques (Birnbrauer and Leach 1993; Farrell et al. 2005). The remaining four studies all reported incorporating some or all aspects of NDBI (Dawson et al. 2010; Haglund et al. 2017; Howard et al. 2005; Rogers et al. 2019), two including the ESDM approach (Dawson et al. 2010; Rogers et al. 2019).

With regard to comparator groups, two of the six unavailable studies contained an eclectic comparator arm, delivered in a school or nursery classroom setting (Farrell et al. 2005; Howard et al. 2005) and four included TAU arms, of which two were delivered in range of settings (Dawson et al. 2010; Rogers et al. 2019; Eikeseth et al. 2007) and two did not provide clear information about setting (Birnbrauer and Leach 1993; Haglund et al. 2017).

**Risk of bias tables**

**Table 8 Risk of bias of studies not included in IPD meta-analyses using the ROBINS-I tool**

| **Study** | **Confounding** | **Selection of Participants** | **Classification of Interventions** | **Deviations from intended interventions** | **Missing data (IPD)** | **Measurement of outcomes** | **Selection of reported result** |
| --- | --- | --- | --- | --- | --- | --- | --- |
| Birnbrauer 1993 | Serious | Moderate | Low | No information | Serious | Moderate | Serious |
| Farrell | Serious | Serious | Low | No information | Serious | Serious | Serious |
| Haglund | No information | No information | No information | No information | No information | No information | No information |
| Howard | Serious | Serious | Low | Moderate | Serious | Serious | Low |

**Table 9 Risk of bias of RCT studies not included in IPD meta-analyses using the Cochrane ROB 2.0tool**

| **Study** | **Randomisation process** | **Deviations from intended interventions** | **Missing outcome data** | **Measurement of outcome** | **Selection of the reported results** | **Overall** |
| --- | --- | --- | --- | --- | --- | --- |
| Dawson 2010/2012 | Low | Low | Low | Some concerns | Low | Some concerns |
| Estes 2015 | Low | Some concerns | High | Some concerns | Some concerns | High |
| Rogers 2019 | Low | Some concerns | Some concerns | Some concerns | Some concerns | Some concerns |

**Meta-analyses figures**


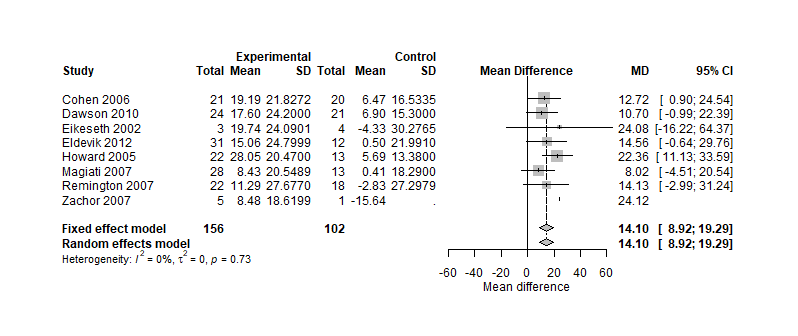


**Fig. 10 Two stage random meta-analysis of IQ at two years, including studies not providing IPD**

**
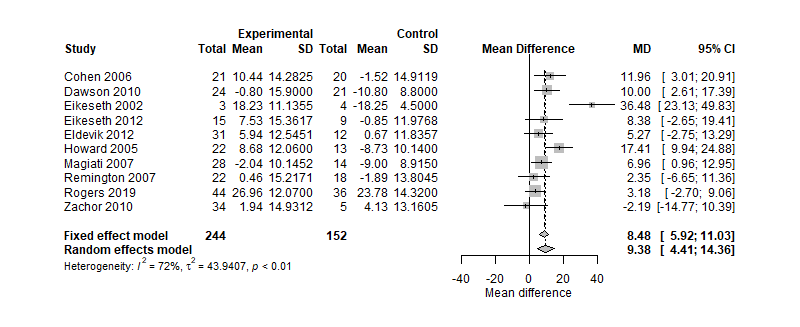
 Fig. 11 Two stage random meta-analysis of composite VABS score at two years, including studies not providing IPD**

Bayley, N. (1969). *Bayley Scales of Infant Development: Birth to Two Years*. New York, USA: Psychological Corporation.

Bayley, N. (1993). *Bayley Scales of Infant Development: Manual*. New York: Psychological Corporation.

Bayley, N. (2006). *Bayley Scales of Infant and Toddler Development–third edition* (Vol. 2). San Antonio, TX, USA: Harcourt Assessment.

Birnbrauer, J. S., & Leach, D. J. (1993). The Murdoch early intervention program after 2 years. *Behaviour Change, 10*(02), 63-74.

Brownell, R. (2000). *Expressive One-Word Picture Vocabulary Test: Manual* (3rd ed.). Novato, California: Academic Therapy Publications.

Conners, C. K. (2001). *Conners' Rating Scales--revised: CRS-R*. North Tonawanda, NJ, USA: Multi-Health Systems.

Dawson, G., Rogers, S., Munson, J., Smith, M., Winter, J., Greenson, J., et al. (2010). Randomized, controlled trial of an intervention for toddlers with autism: the Early Start Denver Model. *Pediatrics, 125*(1), e17-23, doi:10.1542/peds.2009-0958.

Dunn, L. M., Dunn, L. M., Whetton, C., & Burley, J. (1997). *British Picture Vocabulary Scale (BPVS-II)* (2nd ed.). Windsor, Berks: NFER-Nelson.

Edwards, S., Garman, M., Hughes, A., Letts, C., & Sinka, I. (1997). The Reynell Developmental Language Scales III (RDLS). Manual. *International Journal of Language & Communication Disorders, 34*, 151-191.

Eikeseth, S., Smith, T., Jahr, E., & Eldevik, S. (2002). Intensive behavioral treatment at school for 4- to 7-year-old children with autism. A 1-year comparison controlled study. *Behavior Modification, 26*(1), 49-68.

Eikeseth, S., Smith, T., Jahr, E., & Eldevik, S. (2007). Outcome for children with autism who began intensive behavioral treatment between ages 4 and 7: a comparison controlled study. *Behavior Modification, 31*(3), 264-278, doi:10.1177/0145445506291396.

Farrell, P., Trigonaki, N., & Webster, D. (2005). An exploratory evaluation of two early intervention programmes for young children with autism. *Educational and Child Psychology, 22*(4), 29.

Gardner, M. F. (1990). *EOWPVT-R : Expressive One-Word Picture Vocabulary Test, Revised*. Novato, California: Academic Therapy Publications.

Haglund, N., Dahlgren, S., Rastam, M., Gustafsson, P., & Kallen, K. (2017). Improvement of autism symptoms after comprehensive intensive early interventions in a clinical setting. *European Psychiatry, 41*, S129-S129, doi:10.1016/j.eurpsy.2017.01.1940.

Howard, J. S., Sparkman, C. R., Cohen, H. G., Green, G., & Stanislaw, H. (2005). A comparison of intensive behavior analytic and eclectic treatments for young children with autism. *Research in Developmental Disabilities, 26*(4), 359-383, doi:10.1016/j.ridd.2004.09.005.

Lord, C., Risi, S., Lambrecht, L., Cook, E. H., Leventhal, B. L., DiLavore, P. C., et al. (2000). The Autism Diagnostic Observation Schedule—Generic: a standard measure of social and communication deficits associated with the spectrum of autism. *Journal of Autism and Developmental Disorders, 30*(3), 205-223, doi:10.1023/a:1005592401947.

Lord, C., & Rutter, M. (2003). Social Communication Questionnaire (SCQ). <https://www.carautismroadmap.org/social-communication-questionnaire-scq/?print=pdf>. Accessed 1st October 2019.

Mullen, E. M. (1995). *Mullen Scales of Early Learning*. Circle Pines, MN, USA: AGS

Reynell, J., & Huntley, M. (1985). *Reynell Developmental Language Scales—Revised manual* (2nd ed.). Windsor: NFER.

Rogers, S. J., Estes, A., Lord, C., Munson, J., Rocha, M., Winter, J., et al. (2019). A multisite randomized controlled two-phase trial of the Early Start Denver Model compared to treatment as usual. *Journal of the American Academy of Child & Adolescent Psychiatry*, doi:<https://doi.org/10.1016/j.jaac.2019.01.004>.

Roid, G. H., & Pomplun, M. (2012). *The Stanford-Binet Intelligence Scales*: The Guilford Press.

Roid, G. H., & Sampers, J. L. (2004). *Merrill-Palmer-Revised: Scales of Development*. Wood Dale, IL, USA: Stoelting.

Rutter, M., Le Couteur, A., & Lord, C. (2003). *Autism Diagnostic Interview-Revised* (Vol. 29). Los Angeles, CA: Western Psychological Services.

Schopler, E., Reichler, R. J., Bashford, A., Lansing, M. D., & Marcus, L. M. (1990). *Individual assessment and treatment for autistic and developmental disabled children: Psychoeducational profile revised (PEP-R).* (Vol. 1). Austin: Pro-Ed.

Sparrow, S. S., Balla, D. A., Cicchetti, D. V., Harrison, P. L., & Doll, E. A. (1984). *Vineland Adaptive Behavior Scales. Vineland-3* (3rd ed.). London: Pearson Education.

Wechsler, D. (1949). *Wechsler Intelligence Scale for Children*. San Antonio, TX, US: Psychological Corporation.

Wechsler, D. (1974). *Wechsler Intelligence Scale for Children-Revised*. San Antonio, TX, USA: Psychological Corporation.

Wechsler, D. (1989). *WPPSI-R: Wechsler Preschool and Primary Scale of Intelligence-Revised*. San Antonio, TX, USA: Psychological Corporation.

Wechsler, D. (1993). *WISC-III: Wechsler Intelligence Scale for Children*. San Antonio, TX, USA: Psychological Corporation.

Wechsler, D. (2002). *Wechsler Preschool and Primary Scale of Intelligence* (3rd ed.). San Antonio, TX, USA: Psychological Corporation.

Wechsler, D. (2012). *Wechsler Preschool and Primary Scale of Intelligence* (4th ed.). San Antonio, TX, USA: Psychological Corporation.
